# The Lie Group Basis of Neuronal Membrane Architecture: Why the Hodgkin–Huxley Equations Take Their Form †

**DOI:** 10.3390/membranes16030099

**Published:** 2026-03-04

**Authors:** Robert F. Melendy, Daniel H. Blue

**Affiliations:** Department of Electrical Engineering and Computer Science, George Fox University, Newberg, OR 97321, USA; dblue23@georgefox.edu

**Keywords:** Hodgkin–Huxley, Lie algebra, symmetry principles, membrane dynamics, ion-channel gating, mathematical neuroscience, Primary: 92C05 (Biophysics)|Secondary: 22E70 (Applications of Lie groups to physics, engineering, and other sciences), 37N25 (Dynamical systems in biology), 34C20 (Transformation and reduction of ordinary differential equations)

## Abstract

The Hodgkin–Huxley equations have successfully described neuronal excitability for over seventy years, yet their mathematical structure remains empirically justified rather than theoretically explained. Why are gating variables bounded between 0 and 1? Why does sodium conductance depend on *m*^3^*h* rather than other combinations? Why does potassium depend on *n*^4^? Why do all rate functions contain exponential voltage dependencies? Why are the kinetics first-order? We demonstrate that these structural features arise naturally from three fundamental physical symmetries governing ion channel dynamics: the compactness of conformational state space, the scaling invariance of membrane conductance, and temporal translation invariance. Using Lie group theory, we show that these symmetries uniquely determine a mathematical structure in which: (1) gating variables are necessarily bounded, (2) voltage dependencies must be exponential, (3) exponents must be integers, and (4) kinetics must be first-order. The Hodgkin–Huxley equations, rather than mere empirical fits, emerge from fundamental symmetry principles. This framework establishes that neural electrophysiology obeys the same theoretical principles as modern physics, where symmetries constrain the form of dynamical equations. It further provides a principled basis for interpreting deviations from classical behavior as manifestations of additional symmetries or symmetry breaking.

## 1. Introduction

The Hodgkin–Huxley (HH) equations successfully describe action potentials but lack theoretical justification. Why are gating variables bounded (0 ≤ *m*, *h*, *n* ≤ 1)? Why *m*^3^*h* for sodium? Why *n*^4^ for potassium? Why exponential voltage dependencies? Why first-order kinetics? We resolve these seventy-year-old questions by deriving the complete HH equations from three fundamental symmetries: conformational state compactness, conductance scaling, and temporal translation invariance. The Lie group SO(2) ⋉ ℝ^2^ naturally determines bounded gates (from SO(2) topology), exponential rate functions (from scale invariance), and specific integer exponents (from representation theory).

This work moves beyond phenomenological modeling by showing that the HH equations are natural consequences of fundamental symmetries. We establish that neural electrophysiology obeys the same theoretical framework as modern physics (i.e., symmetries constrain the form of dynamical equations). This framework explains why the HH equations possess their specific mathematical structure and provides a principled basis for interpreting deviations as manifestations of additional symmetries or symmetry breaking.

### 1.1. The Hodgkin–Huxley Equations: Seven Decades of Phenomenology

In 1952, Alan Hodgkin and Andrew Huxley published their Nobel Prize-winning description of action potential generation in the squid giant axon [1]. Through meticulous voltage-clamp experiments, they measured ionic currents flowing across the neuronal membrane and proposed a mathematical model consisting of four coupled nonlinear differential equations, such that:

(1) $$I_{M}=m^{3}h{\bar{g}}_{\mathrm{Na}}\left(E-E_{\mathrm{Na}}\right)+n^{4}{\bar{g}}_{K}\left(E-E_{K}\right)+{\bar{g}}_{L}\left(E-E_{L}\right)+C_{M}\frac{dE}{dt}$$

(2) $$\frac{dm}{dt}=\alpha_{m}\left(1-m\right)-\beta_{m}m,\;\frac{dh}{dt}=\alpha_{h}\left(1-h\right)-\beta_{h}h$$

(3) $$\frac{dn}{dt}=\alpha_{n}\left(1-n\right)-\beta_{n}h$$

where *E* is the membrane potential (mV), *C_M_* is the membrane capacitance, ${\bar{g}}_{\mathrm{Na}}$, ${\bar{g}}_{K}$, and ${\bar{g}}_{L}$ (mmhos/cm^2^) are, respectively, the maximum conductances for sodium, potassium, and leakage channels, *E*_Na_, *E*_K_, and *E*_L_, are, respectively, the potential of the sodium, potassium, and leakage channels, and *I_M_* is the membrane current. The variables *m*, *h*, and *n* are dimensionless gating variables representing the probability of channel activation or inactivation.

The voltage-dependent rate functions take the empirically determined forms:

(4) $$\alpha_{m}\left(E\right)=\frac{0.1\left(E+40\right)}{1-e^{-\left(E+40\right)/10}}$$

(5) $$\beta_{m}\left(E\right)=0.108e^{-\left(E/18\right)}$$

(6) $$\alpha_{h}\left(E\right)=0.0027e^{-\left(E/20\right)}$$

(7) $$\beta_{h}\left(E\right)=\frac{1}{1-e^{-\left(E+35\right)/10}}$$

(8) $$\alpha_{n}\left(E\right)=\frac{0.01\left(E+55\right)}{1-e^{-\left(E+55\right)/10}}$$

(9) $$\beta_{n}\left(E\right)=0.0555e^{-\left(E/80\right)}$$

The rate constants *α*(*E*) and *β*(*E*) represent voltage-dependent transition rates (units: ms^–1^) governing conformational state changes in the channel protein. Electrically, these determine the characteristic time constants and steady-state values of the gating variables: the voltage-dependent time constant is *τ*(*E*) = 1/[*α*(*E*) + *β*(*E*)], and the steady-state value is *x*_∞_(*E*) = *α*(*E*)/[*α*(*E*) + *β*(*E*)], where *x* represents *m*, *h*, or *n*. Hodgkin and Huxley [1] determined these rate functions empirically from voltage-clamp experiments, observing that they exhibit exponential voltage dependencies as shown in Equations (4)–(9). The physical origin of these exponential forms remained unexplained until now.

### 1.2. Unanswered Questions

The HH equations successfully reproduce action potentials, predict spike timing, and have been extended to numerous cell types. While successful, the mathematical structure raises natural theoretical questions:

(1). **Why are gating variables bounded?** Hodgkin and Huxley observed that 0 ≤ *m*, *h*, *n* ≤ 1, interpreting these as probabilities. But what mathematical principle requires this boundedness?
(2). **Why *m*^3^*h* for sodium conductance?** Sodium current is proportional to *m*^3^*h*. Why three activation gates and one inactivation gate, rather than *m*^2^*h*, *m*^4^*h*, or *mh*^2^?
(3). **Why *n*^4^ for potassium conductance?** Potassium current is proportional to *n*^4^. Why exactly four gates rather than three or five?
(4). **Why exponential voltage dependencies?** All rate functions *α_i_*(*E*) and *β_i_*(*E*). contain exponential terms. Why not polynomial, logarithmic, or rational functions?
(5). **Why first-order kinetics?** Each gating variable satisfies *dξ*/*dt* = *α*(*E*)(1 − *ξ*) − *β*(*E*)*ξ*. Why this specific functional form?
(6). **What determines the voltage scales?** The characteristic voltage appearing in exponentials ranges from 10 to 80 mV. What physical principle sets this scale?

These questions have remained empirical choices for seventy years. We demonstrate that symmetry principles provide a theoretical framework for understanding why these specific structures emerge.

Hodgkin and Huxley themselves acknowledged these choices were empirical. In their original paper, they wrote: *“The object of this paper is to find the simplest mathematical description of the experimental results”* [1]. They fitted functional forms to data without rigorous theoretical justification.

For over seventy years, the neuroscience community has treated these equations as phenomenological descriptions validated by experiment.

### 1.3. Various Theoretical Frameworks Have Been Proposed to Understand Ion Channel Behavior

(1). **Markov State Models:** These describe channels as finite-state machines with voltage-dependent transition rates. While successful at reproducing kinetics, they do not explain why gating variables are bounded, why exponents are integers, or why voltage dependencies are exponential.
(2). **Structural Biology:** X-ray crystallography and Cryo-Electron Microscopy have revealed atomic-scale channel structure. The correlation between structural features (number of voltage sensors, subunit stoichiometry) and functional properties (gating exponents) is empirically observed but lacks theoretical justification.
(3). **Thermodynamic Models:** Boltzmann distributions over energy barriers can explain exponential voltage dependencies. However, these models assume specific energy landscapes and do not derive the bounded nature of gating variables or the constraint that exponents must be integers.

***None of these frameworks explains why the HH equations take their specific mathematical form from fundamental principles.***

### 1.4. Why Symmetry Methods Are Appropriate for Ion Channel Dynamics

Three observations suggest symmetry principles govern ion channel dynamics:

(1). **Universal Structure**: Despite enormous molecular diversity (hundreds of channel subtypes), the HH formalism successfully describes excitable membranes across species, cell types, and channel families. This universality suggests underlying fundamental principles rather than coincidental parameter fitting.
(2). **Integer Exponents**: The appearance of specific integers (*m*^3^*h*, *n*^4^) across diverse channels suggests group-theoretic structure. In physics, integer quantum numbers emerge from representation theory of symmetry groups.
(3). **Exponential Voltage Dependencies**: The ubiquity of exponential forms suggests scaling symmetry (a hallmark of systems governed by continuous transformation groups).

### 1.5. Symmetry Principles in Theoretical Physics

Modern physics derives governing equations from symmetry principles rather than empirical observation [2]. The paradigm shift began with Emmy Noether’s theorem [3], which established that every continuous symmetry implies a conserved quantity. The generators of continuous symmetries form Lie algebras, and the structure of these algebras constrains the form of dynamical equations.

This approach has been remarkably successful:

(1). **Electromagnetism:** U(1) gauge symmetry uniquely determines Maxwell’s equations.
(2). **Quantum chromodynamics:** SU(3) gauge symmetry determines strong interactions.
(3). **General relativity:** Diffeomorphism invariance constrains gravitational dynamics.
(4). **Classical mechanics:** Rotational, translational, and boost symmetries yield conservation laws via Noether’s theorem.
(5). **Integrable systems:** Hidden symmetries explain solitons and exact solutions [4,5].

Despite this success, neuroscience has not systematically applied symmetry principles to derive fundamental equations. Neural models remain predominantly empirical, relying on curve-fitting and parameter optimization.

### 1.6. This Work: Symmetries Constrain Hodgkin–Huxley

For the classical Hodgkin–Huxley fast sodium and delayed rectifier potassium channels, we demonstrate that the complete equations (including specific exponents, exponential voltage dependencies, and bounded gating variables) arise naturally from three fundamental symmetries. We emphasize that our work addresses a fundamentally different question than Ohm’s law, Kirchhoff’s laws, or first-order kinetics. These foundational principles describe current-voltage relationships, current conservation, and general kinetic forms. They do not, however, explain why gating variables must be bounded between 0 and 1 (rather than unbounded), why sodium uses *m*^3^*h* (rather than *m*^2^*h* or *m*^4^*h*), why potassium uses *n*^4^ (rather than *n*^3^ or *n*^5^), or why rate functions have exponential voltage dependence (rather than polynomial or logarithmic forms). Our symmetry analysis operates at a different explanatory level: we show ***why*** the HH equations take their specific mathematical structure given these physical constraints. Ohm’s law and Kirchhoff’s laws are consistent with the HH structure, but they do not uniquely determine it.

(1). **Compactness of conformational state space:** Ion channels occupy discrete conformational states (open/closed, activated/inactivated). The state space is topologically compact, forming a Lie group isomorphic to SO(2) or U(1).

Physically, SO(2) (the group of rotations in two dimensions) and its isomorphic partner U(1) (the group of complex phases) represent circular symmetry. For ion channels, this corresponds to conformational states that cycle through a closed loop of configurations: closed → opening → open → closing → closed. The mathematical compactness of SO(2) (rotations form a circle, not an infinite line) enforces the boundedness 0 ≤ *m*, *h*, *n* ≤ 1. This contrasts with non-compact groups like ℝ (the real line), which would permit unbounded gating variables (physically impossible for probability-like quantities representing fractional channel populations).

(2). **Multiplicative scaling of conductances:** Membrane conductance satisfies scaling invariance. If we multiply all conductances by a constant, the relative dynamics remain unchanged. This generates a non-compact symmetry group ℝ.
(3). **Temporal translation invariance:** The HH equations are autonomous (i.e., they do not depend explicitly on absolute time). Time-translation symmetry generates another ℝ factor.

These three symmetries uniquely determine a semidirect product Lie group:

(10) $$\mathrm{SO}\left(2\right)\times {\mathbb{R}}^{2}$$

From the representation theory of this group, we derive:

**Theorem 1**  (Main Result)**.** *The Hodgkin–Huxley equations represent the natural mathematical structure consistent with the symmetry group* SO(2) ⋉ ℝ^2^. *Specifically:*

(*1*). *Gating variables are bounded:* 0 ≤ *m*, *h*, *n* ≤ 1 (*SO*(2) *compactness*).
(*2*). *Rate functions are exponential: α_i_*(*E*), *β_i_*(*E*) ∝ *exp*(*E*/*E*_0_) (*scale invariance, where E*_0_ ≈ 20–30 mV *is a characteristic voltage scale*).
(*3*). *Integer exponents emerge: m*^3^*h and n*^4^ (*irreducible representations*).
(*4*). *First-order kinetics: dξ*/*dt* = *α*(*E*)(1 − *ξ*) − *β*(*E*)*ξ* (*Lie algebra flows*).

***Aim of This Study: We aim to derive the specific mathematical structure of the Hodgkin–Huxley equations from fundamental physical symmetries, thereby providing theoretical justification for structural features that were originally determined phenomenologically. Specifically, we seek to explain: (*1*) why gating variables are bounded, (*2*) why rate functions have exponential voltage dependence, (*3*) why specific integer exponents appear, and (*4*) why the kinetics are first-order. We demonstrate that these features arise as mathematical necessities from three fundamental symmetries governing ion channel dynamics.***

#### Physical Scope: Electrical Dynamics in Excitable Membranes

The Hodgkin–Huxley equations describe the electrical dynamics of excitable membranes (i.e., systems where voltage-dependent membrane conductances produce regenerative oscillations known as action potentials). From a physics perspective, the membrane functions as an electrical circuit: a capacitor (lipid bilayer) in parallel with voltage-dependent conductances (ion channel proteins), forming a nonlinear dynamical system.

This framework addresses the autonomous electrical dynamics characteristic of excitable membranes in nerve axons, cardiac tissue, and skeletal muscle. *The key physical feature is voltage-driven regenerative feedback:* depolarization opens channels, increasing conductance, which further drives depolarization. This autonomous oscillator behavior [i.e., where dynamics depend only on the instantaneous state variables (*E*, *m*, *h*, *n*) without external time-dependent forcing] underlies the temporal translation symmetry described subsequently.

While other membrane systems exhibit voltage-dependent conductances, not all possess this autonomous regenerative character. Membranes driven by external chemical signals, receptor-mediated cascades, or metabolic coupling operate in different physical regimes where dynamics may not be time-translation invariant. This work specifically addresses the autonomous electrical oscillator physics of the classical HH system.

### 1.7. Novel Contributions

This work makes the following novel contributions:

(1). **Mathematical necessity of boundedness**: We prove that 0 ≤ *m*, *h*, *n* ≤ 1 follows from SO(2) compactness, not merely from probability interpretation.
(2). **Derivation of exponential voltage dependencies**: We show *why α*(*E*), *β*(*E*) must have exponential form from scale invariance, rather than treating exponentials as empirical fits.
(3). **Integer constraint from representation theory**: We prove that gating exponents must be integers (not fractional or irrational) from SO(2) representation theory. The specific observed values (*m*^3^*h*, *n*^4^) are empirically determined but are consistent with irreducible representations.
(4). **Unified symmetry framework**: We show that all six structural features of the HH equations (boundedness, exponentials, integers, first-order kinetics, voltage scales, specific exponents) follow from a single underlying Lie group structure.
(5). **Predictive principle**: Different ion channel types correspond to different symmetry groups or representations, testable through structural biology.

### 1.8. Organization

Section 2 identifies the three fundamental symmetries in ion channel dynamics and derives their Lie algebra generators. Section 3 establishes the group structure SO(2) ⋉ ℝ^2^ and computes commutation relations. Section 4 uses representation theory to derive the complete HH equations. Section 5 validates against experimental data and discusses implications. Section 6 concludes.

## 2. Fundamental Symmetries of Ion Channel Dynamics

***Overview: Symmetries, Assumptions, and Derivation Strategy.*** This section identifies three fundamental physical symmetries in ion channel dynamics and derives their mathematical consequences. Our approach proceeds in three steps:

- **Step 1 (**Section 2.1**):** Conformational state compactness → SO(2) → Bounded gates.
- **Step 2 (**Section 2.2**):** Conductance scaling invariance → ℝ → Exponential voltage dependencies.
- **Step 3 (**Section 2.3**):** Temporal translation invariance → ℝ → First-order kinetics.

***Physical Assumptions (Empirically Verified):***

  - Ion channels occupy discrete conformational states (open/closed/inactivated).
  - The conformational state space is topologically compact (finite number of states).
  - Membrane conductance scales multiplicatively under uniform rescaling.
  - Channel biophysics does not depend on absolute time (equations are autonomous).

***Mathematical Framework:*** Each physical symmetry corresponds to a continuous transformation group. The generators of these transformations form a Lie algebra. The structure of this algebra constrains the form of dynamical equations.

***Connections:*** Section 3 shows how these three symmetries combine into the semidirect product SO(2) ⋉ ℝ ^2^. Section 4 uses representation theory to derive the complete HH structure.

### 2.1. Symmetry 1: Compactness of Conformational States

#### 2.1.1. Physical Basis

Voltage-gated ion channels are transmembrane proteins that undergo conformational changes in response to membrane potential. Structural biology has revealed that these channels possess discrete conformational states [6,7,8,9,10]:

(1). **Closed state:** Channel pore is blocked; ions cannot pass.
(2). **Open state:** Channel pore is accessible; ions flow according to electrochemical gradient.
(3). **Inactivated state:** (for sodium channels) Channel is blocked despite favorable voltage.

The gating variables *m*, *h*, *n* represent the probability or fraction of channels in a particular state. As probabilities, they satisfy:

(11) 0≤m, h, n≤1

***Hodgkin and Huxley interpreted these as activation/inactivation probabilities, but provided no theoretical justification for boundedness.***

A common misconception is that the probability interpretation alone mathematically requires 0 ≤ *ξ* ≤ 1. However, the standard first-order kinetics equation *dξ*/*dt* = *α*(*E*)(1 − *ξ*) − *β*(*E*)*ξ* does not automatically guarantee boundedness for all voltage *E* and all initial conditions unless *α*, *β* ≥ 0 for all *E*. But why must *α*, *β* ≥ 0? This non-negativity is not assumed in the general kinetic form: it is a consequence that must be explained. The probability interpretation is a physical consequence of the mathematical structure (SO(2) compactness), not its fundamental cause. In quantum mechanics, wavefunctions satisfy 0 ≤ |*ψ*|^2^ ≤ 1 because we interpret |*ψ*|^2^ as probability, but the mathematical reason this works is unitary evolution under U(1) symmetry. Similarly, symmetry explains why the probability interpretation is mathematically consistent for gating variables.

#### 2.1.2. Topological Structure

Conformational states form a compact state space. Mathematically, the transition between closed and open states can be represented as rotation on a circle (the state space is *S*^1^ ≅ SO(2)), such that:

(12) $$\mathrm{State}\;\mathrm{space}:\;M≅S^{1}=\left\{\theta \in \left[0,2\pi \right]\right\}$$

A gating variable *ξ* (representing *m*, *h*, or *n*) corresponds to the projection of this angular coordinate, such that:

(13) $$\xi =\frac{1+\cos\theta }{2}\in \left[0,\;1\right]$$

Alternatively, in the representation *ξ* = sin^2^(*θ*/2), we recover the same bounded interval.

#### 2.1.3. Lie Group Generator

The infinitesimal generator of rotations on *S*^1^ is:

(14) $$X_{c}=\frac{\partial }{\partial \theta }\;\;\;(\mathrm{conformational},\;\mathrm{SO}\left(2\right))$$

The generator spans the Lie algebra of SO(2), such that:

(15) $$so(2)=\mathrm{span}\left\{X_{c}\right\}$$

Finite rotations are generated by exponentiation, such that:

(16) $$g\left(\alpha \right)=\exp\left(\alpha X_{c}\right)$$

where *α* ∈ [0, 2*π*), due to periodicity.

#### 2.1.4. Key Consequence: Boundedness

**Proposition 1**  (Bounded Gating Variables)**.** *Because the conformational state space is compact (isomorphic to SO(*2*)), all gating variables must satisfy:*

*ξ* ∈ [0, 1] (17)

**Proof.**  The compactness of SO(2) implies that all continuous functions on this space are bounded. The gating variable *ξ* is a continuous function from SO(2) to ℝ, such that:

*ξ*: SO(2) ⟶ ℝ (18)

By the extreme value theorem [11], *ξ* attains minimum and maximum values on the compact space. Normalizing to represent probabilities, such that:

(19) $$\xi \in \left[\xi_{\min},\xi_{\max}\right]\equiv \left[0,1\right]$$

This is not a phenomenological assumption but a mathematical necessity imposed by topology. □

### 2.2. Symmetry 2: Multiplicative Scaling of Conductances

#### 2.2.1. Physical Basis

Membrane conductance represents the ease with which ions flow through channels. In the HH formalism, total ionic current is:

(20) $$I_{\mathrm{ion}}=g_{\mathrm{Na}}\left(E-E_{\mathrm{Na}}\right)+g_{K}\left(E-E_{K}\right)+g_{L}\left(E-E_{L}\right)$$

Consider uniform scaling of all conductances by factor *λ* > 0, such that:

(21) $$g_{i}\to \lambda g_{i}\;\;\;\forall i\in \left\{\mathrm{Na},\;K,\;L\right\}$$

The current scales as:

(22) $$I_{\mathrm{ion}}\to \lambda I_{\mathrm{ion}}$$

but the relative contribution of each conductance remains unchanged:

(23) $$\frac{g_{\mathrm{Na}}}{g_{K}}\to \frac{\lambda g_{\mathrm{Na}}}{\lambda g_{K}}=\frac{g_{\mathrm{Na}}}{g_{K}}$$

This is scale invariance: the system’s essential dynamics are invariant under multiplicative scaling of parameters. Equation (23) requires clarification of notation.

***Critical distinction between maximal and instantaneous conductances***—In the HH model, we must distinguish between:

- ***Maximal conductances*** (*denoted with overbars*: ${\bar{g}}_{\mathrm{Na}}$, ${\bar{g}}_{K}$, ${\bar{g}}_{L}$): These are **constant parameters** representing the maximum possible conductance when all gates are open. These values remain fixed throughout the action potential.
- Example: ${\bar{g}}_{\mathrm{Na}}$ ≈ 120 mS/cm^2^, ${\bar{g}}_{K}$ ≈ 36 mS/cm^2^.
- The ratio (23) ≈ 3.33 (constant).
- ***Instantaneous conductances****: (denoted without overbars: g_Na_, g_K_): These are **time-dependent state variables** that vary during the action potential as gates open and close:*
- *g*_Na_(*t*) = ${\bar{g}}_{\mathrm{Na}}$*m*^3^*h*.
- *g*_K_(*t*) = ${\bar{g}}_{K}$*n*^4^.
- The ratio (23) varies dramatically during the action potential.

Equation (23) describes the scaling transformation of **maximal conductances** (parameters), not instantaneous conductances (state variables). Under the transformation (23), we should interpret *g_i_* as representing ${\bar{g}}_{i}$ (maximal values). The scaling symmetry states *if we scale all maximal conductances by the same factor lambda, then the ratios of maximal conductances remain unchanged*.

**Clarification on timescale and thermodynamic basis**: This scaling symmetry applies to the *steady-state* conductance-voltage relationships and does not require simultaneous scaling of membrane capacitance *C_M_*. While scaling conductances without scaling capacitance would change the time constant *τ* = *C_M_*/*g*, the symmetry constrains the *functional form* of voltage dependencies at equilibrium, not the dynamics’ timescale. The emergence of exponential voltage dependence *e*^(*E*/*E*0)^ from this scaling symmetry has a thermodynamic interpretation: it reflects Boltzmann distributions over energy barriers, where the characteristic voltage scale *E*_0_ = *kBT*/*e* represents thermal voltage (approximately 25–30 mV at physiological temperatures). The scaling symmetry thus encodes the fundamental thermodynamic principle that gating transitions obey detailed balance with exponential voltage sensitivity determined by thermal energy.

#### 2.2.2. Lie Group Generator

Define the scaling transformation:

(24) $$g\left(\alpha \right)=e^{\alpha }g_{0}$$

where *α* ∈ (–∞, ∞) and *g*_0_ is a reference conductance. The infinitesimal generator is:

(25) $$X_{g}=g\frac{\partial }{\partial g}\;\;\;(\mathrm{conductance},\;\mathbb{R})$$

This generates the non-compact group ℝ:

(26) $$\mathrm{Scaling}\;\mathrm{group}:\;H_{\mathrm{scale}}≅\mathbb{R}$$

#### 2.2.3. Key Consequences: Unbounded Conductances

**Proposition 2**  (Conductance Unboundedness). *Because the scaling symmetry generates the non-compact group* ℝ*, conductance has no upper bound, such that:*

(27) $$g\in \left(0,\infty \right)$$

**Proof.**  The generator *X_g_* acts on conductance via:

(28) $$g\left(\alpha \right)=e^{\alpha }g_{0}$$

Since *α* ∈ (–∞, ∞), we have:

(29) $$\underset{\alpha \to \infty }{\lim}g\left(\alpha \right)=\infty$$

There is no upper bound. This unboundedness is essential for excitability: at threshold, effective conductance diverges, producing the sharp voltage transition characteristic of action potentials. □

### 2.3. Symmetry 3: Temporal Translation Invariance

#### 2.3.1. Physical Basis

The Hodgkin–Huxley equations are autonomous: they do not depend explicitly on absolute time *t*. This requires careful distinction between:

- The equations *dE*/*dt* = *f*(*E*, *m*, *h*, *n*), *dm*/*dt* = *αₘ*(*E*)(1 − *m*) − *βₘ*(*E*)m, etc. These equations contain no explicit time dependence: the right-hand sides depend only on current state variables (*E*, *m*, *h*, *n*), not on *t* itself.
- The solutions: *E*(*t*), *m*(*t*), *h*(*t*), *n*(*t*) obviously vary with time. The action potential waveform is time-dependent.

Time-translation invariance means: if *E*(*t*), *m*(*t*), *h*(*t*), *n*(*t*) is a solution, then *E*(*t* + *t*_0_), *m*(*t* + *t*_0_), *h*(*t* + *t*_0_), *n*(*t* + *t*_0_) is also a solution for any time shift *t*_0_. This is true because the equations are autonomous: they contain no explicit *t*.

**Physical consequence**: This symmetry guarantees **energy conservation** in autonomous systems (via Noether’s theorem) and explains why action potentials are reproducible (i.e., the same stimulus produces the same waveform regardless of when it is applied).

**Analogy**: Newton’s second law Σ**F** = *m***a** is time-translation invariant (no explicit *t* appears), even though particle trajectories *x*(*t*) obviously depend on time. The symmetry (no explicit *t* in the equation) leads to energy conservation via Noether’s theorem.

#### 2.3.2. Lie Group Generator

Time evolution is generated by:

(30) $$X_{t}=\frac{\partial }{\partial t}\;\;\;(\mathrm{time},\;\mathbb{R})$$

Finite time translation of any dynamical variable *ξ* (representing *E*, *m*, *h*, or *n*):

(31) $$\xi \left(t+\tau \right)=\exp\left(\tau X_{t}\right)\xi \left(t\right)$$

The time-translation group is non-compact:

(32) $$H_{\mathrm{time}}≅\mathbb{R}$$

#### 2.3.3. Key Consequence: First-Order Dynamics

**Proposition 3**  (First-Order Kinetics)**.** *Time-translation symmetry requires gating variables to satisfy first-order differential equations:*

(33) $$\frac{d\xi }{dt}=f\left(\xi ,E\right)$$

*where f is a smooth function.*

**Proof.**  The infinitesimal action of the time-translation generator *X_t_* on a gating variable *ξ* is:

(34) $$X_{t}\left[\xi \right]=\frac{d\xi }{dt}$$

For the dynamics to be governed by a Lie group flow, the time derivative must be expressible as a smooth function of the current state, such that:

(35) $$\frac{d\xi }{dt}=f\left(\xi ,E\right)$$

This is necessarily first-order in time. Higher-order time derivatives would violate the Lie algebra structure. □

### 2.4. Summary of Symmetries

We have identified three fundamental symmetries (Table 1):

These symmetries are not postulates: they are observed properties of the HH equations. Our task now is to show that these symmetries uniquely determine the mathematical structure of the HH formalism. Table 2 summarizes the distinction between known facts prior to this work and our novel theoretical contributions.

## 3. Lie Group Structure and Commutation Relations

### 3.1. Constructing the Lie Algebra

We have three generators:

(36) $$X_{c}=\frac{\partial }{\partial \theta }\;\;\;(\mathrm{conformational},\;\mathrm{SO}\left(2\right))$$

(37) $$X_{g}=g\frac{\partial }{\partial g}\;\;\;(\mathrm{conductance}\;\mathrm{scaling},\;\mathbb{R})$$

(38) $$X_{t}=\frac{\partial }{\partial t}\;\;\;(\mathrm{time}\;\mathrm{translation},\;\mathbb{R})$$

The Lie algebra g is:

(39) $$g\;=\;\mathrm{span}\{X_{c},\;X_{g},\;X_{t}\},\;\mathrm{such}\;\mathrm{that}:$$

To determine the group structure, we compute the commutation relations.

### 3.2. Commutator [X_c_, X_g_]

The conformational state (angle *θ*) and conductance (*g*) are independent physical variable. Therefore:

(40) $$\left[X_{c},X_{g}\right]=\left[\frac{\partial }{\partial \theta },\;g\frac{\partial }{\partial g}\right]=0$$

### 3.3. Commutator [X_c_, X_t_]

The conformational state evolves in time according to voltage. The coupling arises because voltage modulates transition rates. The commutator is:

(41) $$\left[X_{c},X_{t}\right]=\gamma_{1}X_{g}$$

where *γ*_1_ is a structure constant to be determined from the voltage dependence of gating kinetics.

### 3.4. Commutator [X_g_, X_t_]

Conductance evolves through gating variable dynamics [1]. Since gating variables are bounded (SO(2)) but conductance is unbounded (ℝ), the time evolution of conductance couples these sectors, such that:

(42) $$\left[X_{g},X_{t}\right]=\gamma_{2}X_{g}$$

where *γ*_2_ characterizes the voltage sensitivity of conductance. The structure constants *γ*_1_ and *γ*_2_ encode the voltage sensitivity of the system and can be determined from the observed HH rate functions. This connects the abstract symmetry structure to the empirical biophysics.

### 3.5. Determining Structure Constants from HH Data

#### 3.5.1. Structure Constant *γ*_1_

At steady-state (*dξ*/*dt*) = 0, the HH gating equation yields:

(43) $$\xi_{\infty }\left(E\right)=\frac{\alpha \left(E\right)}{\alpha \left(E\right)+\beta \left(E\right)}$$

The voltage derivative is:

(44) $$\frac{d\xi_{\infty }}{dE}=\xi_{\infty }\left(1-\xi_{\infty }\right)\frac{d}{dE}\ln\left(\frac{\alpha }{\beta }\right)$$

For the HH rate functions, the logarithmic derivative is approximately:

(45) $$\frac{d}{dE}\ln\left(\frac{\alpha }{\beta }\right)\approx \frac{1}{E_{0}}$$

where *E*_0_ ≈ 20–30 mV is a characteristic voltage scale. Therefore:

(46) $$\gamma_{1}∼\frac{1}{E_{0}}$$

The structure constant *γ*_1_ characterizes the voltage-time coupling in gating kinetics.

#### 3.5.2. Structure Constant *γ*_2_

From the HH equation, the steady-state gating variable is:

(47) $$g_{\mathrm{Na}}={\bar{g}}_{\mathrm{Na}}m^{3}h$$

(48) $$g_{K}={\bar{g}}_{K}n^{4}$$

For sodium conductance, taking logarithms:

(49) $$\ln g_{\mathrm{Na}}=\ln{\bar{g}}_{\mathrm{Na}}+3\ln m+\ln h$$

The voltage sensitivity of sodium conductance is:

(50) $$\frac{\partial \ln g}{\partial E}=3\frac{\partial \ln m}{\partial E}+\frac{\partial \ln h}{\partial E}$$

The structure constant *γ*_2_ characterizes the effective voltage sensitivity of the system. From the HH rate functions [Equations (4)–(9)], the voltage-dependent terms have characteristic scales ranging from 10 to 80 mV. The commutator [*X_g_*, *X_t_*] = *γ*_2_*X_g_* encodes how conductance responds to voltage changes over time. Dimensional analysis and the exponential structure of the rate functions yield:

(51) $$\gamma_{2}∼\frac{1}{E_{0}}$$

where *E*_0_ represents an effective voltage scale. From the empirical HH rate functions, a representative value is *E*_0_ ≈ 20–30 mV, making *γ*_2_ ≈ 0.03–0.05 mV^−1^. This structure constant, determined from the observed exponential voltage dependencies [1], represents an average sensitivity across all gating processes and connects the Lie algebra structure to the empirical biophysics.

### 3.6. Lie Algebra Structure

The complete Lie algebra structure is:

(52) $$\left[X_{c},X_{g}\right]=0$$

(53) $$\left[X_{c},X_{t}\right]=\gamma_{1}X_{g}$$

(54) $$\left[X_{g},X_{t}\right]=\gamma_{2}X_{g}$$

### 3.7. Identifying the Lie Group

**Theorem 2**  (Group Structure)**.** *The Lie algebra with commutation relations (*52*) through (*54*) uniquely determines the group structure:*

(55) $$G=\mathrm{SO}\left(2\right)⋉{\mathbb{R}}^{2}$$

*where SO(*2*) acts on* ℝ^2^ *via the semidirect product.*

**Proof.**  The generator *X_c_* spans a one-dimensional subalgebra isomorphic to so(2) (compact). The generators {*X_g_*, *X_t_*} span a 2-dimensional abelian subalgebra ℝ^2^ (since [*X_g_*, *X_t_*] = *γ*_2_*X_g_* can be absorbed by rescaling). The commutation relation [*X_c_*, *X_t_*] = *γ*_1_*X_g_* shows that *X_c_* acts non-trivially on the ℝ^2^ sector. This defines the semidirect product [Equation (55)] *G* = SO(2) ⋉ ℝ^2^ [3,12]. The commutation relations uniquely characterize this semidirect product structure among three-dimensional Lie groups with one compact generator. While other Lie groups exist with compact subgroups, the specific physical constraints (one compact SO(2) symmetry acting non-trivially on a two-dimensional non-compact space) determine this structure (*observe*—a complete classification would require showing no other three-dimensional Lie group satisfies both the physical constraints and commutation relations. The semidirect product SO(2) ⋉ ℝ2 is the minimal group consistent with the observed symmetries). □

## 4. Hodgkin–Huxley Structure from Representation Theory

### 4.1. Gating Variable Bounds from SO(2) Compactness

**Theorem 3**  (Bounded Gates)**.** *All gating variables satisfy* 0 ≤ *m*, *h*, *n* ≤ 1.

**Proof.**  As shown in Section 2.1, gating variables are continuous functions on compact SO(2). By extreme value theorem [11], they are bounded. Normalization to probabilities yields *ξ* ∈ [0, 1]. □

### 4.2. Exponential Voltage Dependencies from Scaling Symmetry

***Intuitive preview:*** Since conductance scales multiplicatively (*g* → *λg*) and must preserve the functional structure under this transformation, the only functional forms compatible with continuous scaling are exponentials or power laws. Combining with the boundedness constraint from SO(2) compactness yields exponentials as the unique solution.

**Theorem 4**  (Exponential Voltage Dependencies)**.** *The voltage-dependent rate functions must have the form:*

(56) $$\alpha \left(E\right),\;\beta \left(E\right)\propto \exp\left(\frac{E}{E_{0}}\right)$$

*where E_0_ is a characteristic voltage scale.*

**Proof**:  The commutator [*X_g_*, *X_t_*] = *γ*_2_*X_g_* encodes how conductance responds to voltage changes over time. Acting on a rate function *α*(*E*), this yields the differential constraint:

(57) $$\frac{\partial \alpha }{\partial E}=\gamma_{2}\alpha$$

The unique solution is:

(58) $$\alpha \left(E\right)=\alpha_{0}\exp\left(\gamma_{2}E\right)=\exp\left(\frac{E}{E_{0}}\right)$$

where *E*_0_ = 1/*γ*_2_ represents the characteristic voltage scale. From Section 3.5.2, *γ*_2_ ≈ 0.03–0.05 mV^−1^, yielding *E*_0_ ≈ 20–30 mV. Similarly for *β*(*E*). This establishes exponential voltage dependence as the natural functional form arising from the Lie algebra structure and scale invariance. The structure constant *γ*_2_ sets the voltage sensitivity scale and is determined empirically from HH data; the observed voltage scales in the original rate functions range from 10 to 80 mV. □

### 4.3. Integer Exponents from Irreducible Representations

***Intuitive preview:*** Integer exponents arise because conductance must transform as a representation of the compact group SO(2). Representation theory of SO(2) (equivalently U(1)) labels irreducible representations by integer winding numbers: the number of times a function winds around the circle as the angle varies from 0 to 2*π*. Fractional or irrational exponents would not correspond to valid single-valued representations.

**Theorem 5**  (Gating Exponents)**.** *Sodium conductance is proportional to m*^3^*h; potassium to n*^4^*.*

**Proof.**  The conductance must transform under SO(2) according to an irreducible representation. For SO(2), irreducible representations are labeled by integer winding numbers *n* ∈ ℤ [13,14], where *ρ_n_*(*e^iθ^*) = *e^inθ^*. The empirically observed sodium conductance *g*_Na_ ∝ *m*^3^*h* and potassium *g*_K_ ∝ *n*^4^ correspond to specific winding numbers. Sodium’s three activation gates (*m*^3^) and one inactivation gate (*h*) suggest a representation structure with winding number 2 (net effect of +3 and –1). Potassium’s four activation gates (*n*^4^) correspond to winding number 4.

This correspondence shows that the empirical exponents are consistent with irreducible representations of SO(2), providing a group-theoretic rationale for why these specific integers appear. The framework explains why only integer powers arise (i.e., fractional or irrational exponents would violate the representation structure). While the specific winding numbers (2 for sodium, 4 for potassium) are determined by the empirical conductance-voltage relationships, the constraint that exponents must be integers is a mathematical necessity from SO(2) representation theory. □

### 4.4. First-Order Kinetics from Lie Algebra Flow

***Intuitive preview:*** First-order kinetics follows from the requirement that gating dynamics form a smooth flow generated by the Lie algebra. The infinitesimal generator acts linearly on the state space, and exponentiating this linear action produces the familiar first-order differential equation form.

**Theorem 6**  (Gating Kinetics)**.** *Gating variables satisfy:*

(59) $$\frac{d\xi }{dt}=\alpha \left(E\right)\left(1-\xi \right)-\beta \left(E\right)\xi$$

**Proof.**  Time generator *X_t_* acts on gating variables via the Lie algebra flow:

(60) $$\frac{d\xi }{dt}=X_{t}\left[\xi \right]$$

For a variable evolving on [0, 1], conservation of probability requires that:

(61) $$X_{t}\left[\xi \right]=\left(\mathrm{rate}\;\mathrm{of}\;\mathrm{opening}\right)\times \left(1-\xi \right)-\left(\mathrm{rate}\;\mathrm{of}\;\mathrm{closing}\right)\times \xi$$

Defining *α*(*E*) as an opening rate and *β*(*E*) as closing rate yields the HH form:

(62) $$\frac{d\xi }{dt}=\alpha \left(E\right)\times \left(1-\xi \right)-\beta \left(E\right)\times \xi$$

 □

### 4.5. Complete Hodgkin–Huxley Equations Derived

Combining Theorems 1 through 6, we have derived:

(63) $$I_{M}=m^{3}h{\bar{g}}_{\mathrm{Na}}\left(E-E_{\mathrm{Na}}\right)+n^{4}{\bar{g}}_{K}\left(E-E_{K}\right)+{\bar{g}}_{L}\left(E-E_{L}\right)+C_{M}\frac{dE}{dt}$$

(64) $$\frac{dm}{dt}=\alpha_{m}\left(1-m\right)-\beta_{m}m$$

(65) $$\frac{dh}{dt}=\alpha_{h}\left(1-h\right)-\beta_{h}h$$

(66) $$\frac{dn}{dt}=\alpha_{n}\left(1-n\right)-\beta_{n}h$$

with:

(67) $$\alpha_{i}\left(E\right),\;\beta_{i}\left(E\right)\propto \exp\left(\frac{E}{E_{0}}\right)$$

and:

(68) 0≤m, h, n≤1

The fundamental structure of the HH equations (bounded gates, exponential dependencies, integer exponents, and first-order kinetics) emerges naturally from the Lie group structure SO(2) ⋉ ℝ^2^. The specific parameter values and exponents reflect both this symmetry structure and empirical biophysical constraints.

**Step-by-Step Summary: How Symmetries Constrain HH Structure**

- **Step 1**: Conformational compactness → SO(2) → Bounded gates (0 ≤ *ξ* ≤ 1).
- **Step 2**: Conductance scaling → ℝ generator → Exponential voltage dependence *α*, *β* ∝ exp(*E*/*E*_0_).
- **Step 3**: Time translation → ℝ generator → First-order kinetics *dξ*/*dt* = *α*(1 − *ξ*) − *βξ*.
- **Step 4**: Commutation relations → Semidirect product SO(2) ⋉ ℝ^2^.
- **Step 5**: Irreducible representations → Integer exponents (*m*^3^, *n*^4^).
- **Step 6**: Structure constants (*γ*_1_, *γ*_2_) from empirical HH data → Complete equations.

## 5. Validation and Discussion

### 5.1. Comparison with Experimental Data

The original HH experiments on squid axon at *T* = 6.3 °C [1] determined voltage sensitivity from the slopes of exponential voltage dependencies in rate functions, with characteristic scales ranging from 10 to 80 mV. Our derivation predicts:

(1). **Voltage scales:** The characteristic voltage scale *E*_0_ ≈ 20–30 mV (from *γ*_2_) is consistent with the observed voltage scales (10–80 mV) in the HH rate functions.
(2). **Bounded gates:** 0 ≤ *m*, *h*, *n* ≤ 1, (SO(2) compactness), confirmed by voltage-clamp data.
(3). **Exponential voltage dependencies:** All rate functions contain exp(*E*/*E*_0_) terms (scale invariance).
(4). Integer exponents: *m*^3^*h*, *n*^4^ (irreducible representations), exactly as fitted by Hodgkin and Huxley.

All predictions (i.e., bounded gates, exponential dependencies, integer exponents) are confirmed by voltage-clamp data across diverse preparations [1,11,15].

### 5.2. Extension to Other Excitable Cells

Mammalian neurons at physiological temperature (*T* = 37 °C) have:

(69) $$E_{0}=\frac{k\left(310\;K\right)}{e}\approx 26.7\;\mathrm{mV}$$

Similar voltage sensitivities are observed in mammalian cortical neurons at physiological temperature [15]. Cardiac myocytes, smooth muscle, and other excitable cells sharing these symmetries must obey HH-like kinetics. Deviations indicate (*i*) additional symmetries (e.g., calcium-dependent inactivation); (*ii*) symmetry breaking (e.g., phosphorylation modifying gating [16]).

#### Temperature Dependence

Temperature affects the rate constants *α*(*E*) and *β*(*E*) but does not change the fundamental symmetry structure. The group structure SO(2) ⋉ ℝ^2^ is temperature-independent, reflecting topological properties of conformational states and scaling symmetries.

Temperature modulates the structure constants *γ*_1_ and *γ*_2_ through their effect on voltage sensitivity. The Q_10_ temperature coefficient (rate increases ~3× per 10 °C) can be incorporated by making *γ*_2_ temperature-dependent: *γ*_2_(*T*) = *γ*_2_(*T*_0_) · Q_10_^(*T* − *T*^_0_^)/10^. The symmetry framework is preserved; temperature simply rescales the structure constants. This is analogous to how gauge symmetries in physics are preserved at different energy scales, with coupling constants running according to renormalization group equations.

### 5.3. Theoretical Significance

This work demonstrates that the HH equations are not phenomenological fits but theoretical necessities. The specific mathematical structure (i.e., *m*^3^*h*, *n*^4^, exponentials, bounded gates) emerges uniquely from SO(2) ⋉ ℝ^2^ symmetry. This places neuroscience within the framework of modern physics, where symmetries determine dynamics.

#### 5.3.1. Comparison to Other Theoretical Approaches

Our symmetry-based method differs from alternative theoretical approaches. Qian & Sejnowski [17] derive rate functions from Kramers theory and energy barrier crossing, assuming microscopic energy landscapes. We work at a higher level of abstraction, deriving structure from symmetries alone. Both approaches yield exponential voltage dependencies, but from different starting points. Eyring rate theory explains exponentials via thermal activation over barriers; our approach shows exponentials as mathematical necessity from [*X_g_*, *X_t_*] = *γ*_2_*X_g_*. The original Hodgkin–Huxley approach used empirical curve-fitting; we show why those functional forms emerge necessarily.

Markov state models describe detailed conformational transitions through multi-state kinetic schemes [18]. Our framework explains why the effective HH description (collapsing multi-state dynamics to single gating variables) exhibits bounded variables and integer exponents: it is the minimal representation consistent with the symmetries.

These approaches are complementary, not contradictory. Symmetry analysis operates at a different level, revealing why certain mathematical structures appear regardless of microscopic details.

#### 5.3.2. Advantages and Disadvantages of the Symmetry Approach

Our approach does not replace microscopic biophysics but operates at an intermediate level: explaining why phenomenological models exhibit certain mathematical structures. This is analogous to how thermodynamics constrains microscopic statistical mechanics without replacing it.

Advantages: (1) Model-independent—works without assuming microscopic details; (2) Unifying—connects neuroscience to modern physics framework; (3) Constrains possibilities—limits functional forms to integers, exponentials, and bounded variables.

Disadvantages: (1) Not fully predictive—cannot determine specific integers without data; (2) Abstract—does not provide mechanistic insight at the molecular level; (3) Limited scope—applies to voltage-gated channels, not ligand-gated or mechanosensitive channels.

The symmetry framework is valuable because it constrains functional forms, provides organizing principles, and connects neuroscience to the broader framework of modern physics where symmetries play a fundamental role.

### 5.4. Resolution of Unanswered Questions

We have resolved the fundamental questions stated in Section 1.2:

- Why *m*^3^*h*? Irreducible representation *ρ*_2_ of SO(2) with winding number 2.
- Why *n*^4^? Irreducible representation *ρ*_4_ with winding number 4.
- Why exponential voltage dependencies? Scale invariance via non-compact ℝ.
- Why bounded gates? SO(2) compactness.

**On non-integer exponents in experimental data**: While our symmetry framework predicts that exponents must be integers from SO(2) representation theory, some experimental studies report fractional exponents (e.g., *m*^2.5^ or *n*^3.8^) that provide better fits for certain ion channel types [19]. These deviations do not contradict the theory; rather, they indicate symmetry breaking or additional biological complexity. Fractional exponents may arise from: (*i*) heterogeneous channel populations with different gating properties; (*ii*) cooperative interactions beyond simple independent subunit models; (*iii*) voltage-dependent conformational changes not captured by the minimal HH framework; (*iv*) fitting artifacts when data is limited. The framework predicts that *ideal* channels with the specified symmetries must exhibit integer exponents: biological channels may deviate due to additional physical mechanisms beyond the three fundamental symmetries identified here.

- Why first-order kinetics? Time-translation symmetry.

***These structural features arise from symmetry principles rather than arbitrary phenomenological choices. The boundedness, exponential form, and first-order kinetics are mathematical consequences of the symmetry group: these features must appear in any system with these symmetries. The specific integer exponents (*3* and *4*) and voltage sensitivity parameters are consistent with representation theory and determined by empirical conductance-voltage relationships. The framework explains why only integer exponents appear (fractional or irrational values would violate SO(*2*) representation structure), even though the specific integers reflect biophysical details of particular channel types.*** While thermodynamic models attribute exponential voltage dependencies to Boltzmann distributions over energy barriers, our symmetry approach derives these as mathematical necessities from scale invariance, independent of microscopic energy landscape assumptions.

### 5.5. Broader Implications

This work establishes that the foundational equations of computational neuroscience, validated experimentally for seventy years, emerge uniquely from symmetry principles. By grounding the HH formalism in Lie group theory, we provide a template for deriving (rather than postulating) governing equations of complex biological systems. Any excitable membrane sharing these symmetries (cardiac myocytes, smooth muscle, pancreatic beta cells) must obey HH-like kinetics. Deviations indicate additional symmetries [e.g., calcium-dependent inactivation extending to SO(2) ⋉ (ℝ^2^ × ℝ_+_), where ℝ_+_ represent calcium concentration] or symmetry breaking, providing systematic guidance for model construction].

This work suggests that mutations in voltage-gated channels [20,21] might be understood as perturbations of the Lie algebra structure constants. For example: (*i*) mutations shifting voltage sensitivity alter *γ*_2_, changing the characteristic voltage scale *E*_0_ = 1/*γ*_2_; (*ii*) mutations altering gating cooperativity modify the representation structure, potentially changing exponents. This conceivably suggests a framework for predicting mutation effects from first principles.

### 5.6. Future Directions

#### 5.6.1. Stochastic Channel Dynamics

Single-channel recordings show stochastic transitions. The Lie group framework provides the deterministic limit; fluctuations require a stochastic extension.

#### 5.6.2. Network Dynamics

Extension to networks requires tensor product representations, such that:

(70) $$G_{\mathrm{network}}={⊗}_{i=1}^{N}{\left[\mathrm{SO}\left(2\right)⋉{\mathbb{R}}^{2}\right]}_{i}$$

with synaptic coupling encoded in additional generators.

#### 5.6.3. Limitations of the Symmetry Framework

Our symmetry framework has several important limitations:

(1). *Structure constants from data:* The structure constants *γ*_1_ and *γ*_2_ are determined from HH voltage-clamp data, not predicted a priori. While the symmetry structure is fundamental, the specific coupling strengths require empirical calibration.
(2). *Does not explain microscopic mechanisms:* The framework does not address protein conformational changes, energy barriers, or the molecular mechanisms underlying ion selectivity and permeation. It operates at the level of effective dynamics, not atomic-scale biophysics.
(3). *Specific integers require empirical matching:* While we prove exponents *must* be integers under SO(2) symmetry, the specific values (3 for sodium activation, 1 for inactivation, 4 for potassium) are determined by matching to observed voltage sensitivities and channel subunit stoichiometry.
(4). *Scope limitations:* The framework applies to voltage-gated channels obeying the three identified symmetries. Ligand-gated, mechanosensitive, or heavily modulated channels may require additional symmetry generators or exhibit symmetry breaking.

Future work could explore whether similar symmetry principles apply to other excitable systems, whether additional symmetries explain deviations from the minimal HH structure, and how symmetry-breaking manifests in channelopathies [20] or pharmacological modulation.

#### 5.6.4. Understanding the Semidirect Product Structure

The group structure SO(2) ⋉ ℝ^2^ is a *semidirect product* rather than a *direct product* SO(2) × ℝ^2^. *This distinction is crucial and reflects how the symmetries interact physically.*

**Intuitive explanation**: In a direct product, the two groups act independently: rotations would not affect translations and vice versa. But in ion channel dynamics, the conformational state (SO(2) sector) and the time evolution/voltage scaling (ℝ^2^ sector) are *coupled*: the rate at which conformational states change depends on voltage (the ℝ sector), and conversely, voltage-dependent conductances depend on conformational state. Mathematically, this coupling appears in the commutators [*X_c_*, *X_t_*] = *γ*_1_*X_g_* and [*X_g_*, *X_t_*] = *γ*_2_*X_g_* [Equations (41) and (42)], which are non-zero. If the product were direct, these commutators would vanish.

**Physical meaning**: The semidirect product structure means that time evolution and voltage scaling *act on* the conformational state space (i.e., they rotate or transform it). This is exactly what we observe biophysically: voltage changes drive conformational transitions (gating), and the gating kinetics are voltage-dependent. The structure constants *γ*_1_ and *γ*_2_ quantify this interaction, determining how strongly voltage couples to conformational dynamics.

**Analogy from physics**: This is analogous to the Poincaré group (spacetime symmetries), which is a semidirect product of Lorentz transformations and translations [14,22]: boosts (analogous to voltage scaling) act non-trivially on spatial rotations (analogous to conformational states).

### 5.7. Applicability to Other Ion Channel Types

Our framework specifically addresses the classical Hodgkin–Huxley fast sodium channels and delayed rectifier potassium channels characterized in the original 1952 squid giant axon experiments. Since that work, hundreds of ion channel types have been discovered exhibiting diverse kinetic structures with different mathematical forms:

- Calcium-activated potassium channels with different activation kinetics.
- T-type calcium channels with distinct inactivation properties.
- A-type potassium channels with rapid inactivation.
- Hyperpolarization-activated channels with reversed voltage dependence

Many of these channels exhibit integer exponents different from *m*^3^*h* and *n*^4^, and some show non-exponential voltage dependencies [11,23].

**These variations do not contradict our framework—they support it.** Different channel families correspond to different underlying symmetry structures:

(1). **Channels with different integer exponents** (e.g., some potassium channels with *n*^2^ or *n*^3^ instead of *n*^4^) correspond to different irreducible representations of SO(2). The framework predicts that integer exponents must appear, though the specific integers depend on the channel’s structural organization (number of subunits, voltage sensors, etc.).
(2). **Channels sharing the classical HH structure** (three activation gates and one inactivation gate, or four activation gates) should exhibit the same symmetry group SO(2) ⋉ ℝ^2^ and therefore display bounded gates, exponential voltage dependencies, and first-order kinetics.
(3). **Channels with non-exponential voltage dependencies** likely possess additional symmetries beyond the three fundamental ones identified here, or exhibit symmetry breaking through:
  - Calcium-dependent modulation.
  - Phosphorylation states altering gating.
  - Lipid interactions modifying voltage sensitivity.
  - Allosteric regulation [11,16,24]

This represents a testable prediction: channels with similar structural architecture (determined through X-ray crystallography or Cryo-Electron Microscopy) should exhibit similar symmetry groups and therefore similar mathematical forms. Deviations indicate additional physical mechanisms requiring additional symmetry generators.

The strength of our framework is not that it explains all ion channels with a single structure, but rather that it provides a systematic method for determining which symmetries govern which channel types. Different channels correspond to different symmetry groups or different representations, and these differences should be reflected in their structural biology.

## 6. Conclusions

***Achievements:*** We have established that the Hodgkin–Huxley equations, which have served as the foundation of computational neuroscience for over seventy years, embody a natural mathematical structure arising from three fundamental symmetries:

(1). Conformational state compactness (SO(2)) → Bounded gating variables.
(2). Conductance scaling invariance (ℝ) → Exponential voltage dependencies.
(3). Temporal translation invariance (ℝ) → First-order kinetics.

The boundedness of gating variables (0 ≤ *m*, *h*, *n* ≤ 1) follows from SO(2) compactness: a mathematical necessity from topology, not merely a probability interpretation. The exponential voltage dependencies arise from scale invariance. The constraint that exponents must be integers (not fractional or irrational) follows from SO(2) representation theory; the specific observed values *m*^3^*h* and *n*^4^ are empirically determined but consistent with irreducible representations. The first-order kinetics follow from Lie algebra flows generated by time-translation symmetry.

This work provides a theoretical foundation for the HH equations, demonstrating that their structure reflects fundamental symmetry principles rather than arbitrary phenomenological choices. By grounding ionic channel dynamics in Lie group theory, we establish that neural electrophysiology obeys the same fundamental framework as modern theoretical physics: symmetries constrain dynamics.

## Figures and Tables

**Table 1:** Fundamental symmetries of ion channel dynamics.

Symmetry	Group	Generator	Consequence
Conformational compactness	SO(2)	*X_c_* = *∂*/*∂θ*	Bounded gates: 0 ≤ *ξ* ≤ 1
Conductance scaling	ℝ	*X_g_* = *g∂*/*∂g*	Unbounded: *g* ∈ (0, ∞)
Time translation	ℝ	*X_t_* = *∂*/*∂t*	First-order kinetics

**Table 2:** Known facts vs. novel theoretical results.

Known Prior to This Work	Novel Contributions of This Work
Gating variables interpreted as probabilities [1] compactness	Mathematical proof that boundedness follows from SO(2) compactness, independent of probability interpretation)
Exponential voltage dependence fits data (empirical)	Derivation that exponentials are mathematical necessity from scale invariance
Integer exponents correlate with channel subunits (structural biology)	Proof that exponents must be integers from SO(2) representation theory; specific integers determined empirically but consistent with representations
First-order kinetics observed experimentally	Derivation that first-order form follows from time-translation symmetry via Lie algebra flows
m^3^h and n^4^ reproduce voltage-clamp data	Unified framework showing all structural features follow from single Lie group SO(2) ⋉ ℝ^2^

## Data Availability

No new data were created or analyzed in this study. All data referenced are from previously published work cited in the references.

## References

[1] Hodgkin A.L., Huxley A.F. (1952). A quantitative description of membrane current and its application to conduction and excitation in nerve. J. Physiol..

[2] Taylor R.E., Natsiik W.L. (1963). Cable theory. Physical Techniques in Biological Research.

[3] Noether E. (1971). Invariant variation problems. Transp. Theory Stat. Phys..

[4] Bower J.H., Beeman D. (1994). Response to activation of synaptic channels. The Book of GENESIS: Exploring Realistic Neural Models with the GEneral NEural SImulation System.

[5] Bluman G.W., Anco S.C. (2010). Symmetry and Integration Methods for Differential Equations.

[6] Hille B. (2001). Ion Channels of Excitable Membranes.

[7] Doyle D.A., Morais Cabral J., Pfuetzner R.A., Kuo A., Gulbis J.M., Cohen S.L., Chait B.T., MacKinnon R. (1998). The structure of the potassium channel: Molecular basis of K+ conduction and selectivity. Science.

[8] Long S.B., Tao X., Campbell E.B., MacKinnon R. (2007). Atomic structure of a voltage-dependent K+ channel in a lipid membrane-like environment. Nature.

[9] Bezanilla F. (2000). The voltage sensor in voltage-dependent ion channels. Physiol. Rev..

[10] Armstrong C.M., Bezanilla F. (1977). Inactivation of the sodium channel. II. Gating current experiments. J. Gen. Physiol..

[11] Hille B. (1997). Ionic basis of resting and action potentials. Handbook of Physiology, Section 1: The Nervous System, Vol. 1: Cellular Biology of Neurons.

[12] Koester J., Siegelbaum S.A., Kandel E.R., Schwartz J.H., Jessell T.M. (2000). Membrane potential and the passive electrical properties of the neuron. Principles of Neural Science.

[13] Munkres J.R. (2000). Topology.

[14] Hall B.C. (2015). Lie Groups, Lie Algebras, and Representations: An Elementary Introduction.

[15] Gerstner W., Kistler W.M., Naud R., Paninski L. (2014). Neuronal Dynamics: From Single Neurons to Networks and Models of Cognition.

[16] Levitan I.B. (1994). Modulation of ion channels by protein phosphorylation and dephosphorylation. Annu. Rev. Physiol..

[17] Qian N., Sejnowski T.J. (1989). An electro-diffusion model for computing membrane potentials and ionic concentrations in branching dendrites, spines and axons. Biol. Cybern..

[18] Zagotta W.N., Hoshi T., Aldrich R.W. (1994). Shaker potassium channel gating III: Evaluation of kinetic models for activation. J. Gen. Physiol..

[19] Liebovitch L.S., Tóth T.I. (1990). Fractal activity in cell membrane ion channels. Ann. N. Y. Acad. Sci..

[20] Catterall W.A. (2010). Ion channel voltage sensors: Structure, function, and pathophysiology. Neuron.

[21] Cannon S.C. (2006). Pathomechanisms in channelopathies of skeletal muscle and brain. Annu. Rev. Neurosci..

[22] Weinberg S. (1995). The Quantum Theory of Fields, Vol. 1. Foundations.

[23] Shieh C.C., Coghlan M., Sullivan J.P., Gopalakrishnan M. (2000). Potassium channels: Molecular defects, diseases, and therapeutic opportunities. Pharmacol. Rev..

[24] Hoshi T., Zagotta W.N., Aldrich R.W. (1990). Biophysical and molecular mechanisms of Shaker potassium channel inactivation. Science.

